# Editorial: Psychological well-being, cognitive demands, and motor control in esports athletes and recreational gamers

**DOI:** 10.3389/fpsyg.2026.1999332

**Published:** 2026-10-01

**Authors:** Philip X. Fuchs, Andrea Fusco, Cristina Cortis, Valeria Saladino

**Affiliations:** 1 Department of Physical Education and Sports Sciences, National Taiwan Normal University, Taipei, Taiwan; 2 Department of Medicine and Aging Sciences, University “G. d'Annunzio” of Chieti-Pescara, Chieti, Italy; 3 Department of Human Sciences, Society and Health, University of Cassino and Lazio Meridionale, Cassino, Italy; 4 Department of Health Sciences, “Magna Graecia” University of Catanzaro, Catanzaro, Italy

**Keywords:** competitive performance, human performance, mental health, neurodiversity, player development, psychophysiology, resilience, self-efficacy

## 1 Introduction and relevance

Video gaming extends from recreation to organized competition and professional performance. At the high-performance end, esports places substantial cognitive-motor demands on players, including attention, rapid decision-making, cognitive flexibility, and sensorimotor control under pressure (Lachowicz et al., 2026; Pedraza-Ramirez et al., 2025). Competitive gaming also produces measurable cardiovascular and autonomic responses, showing that sedentary play can impose relevant psychophysiological demands (Ketelhut and Nigg, 2024; Lyu et al., 2026). These performance demands occur alongside stress, motivation, resilience, sleep, and social processes (Giakoni-Ramírez et al., 2022; Kegelaers et al., 2024; Poulus et al., 2024).

The scale and increasing institutionalization of esports (Kegelaers et al., 2024) make these issues relevant beyond professional competitors. Mental-health concerns are prominent in esports research, although evidence does not indicate uniformly poorer mental-health or sleep outcomes among esports participants than non-participants (Poulus et al., 2026; Tang et al., 2026). Extended sitting, screen exposure, and demanding training schedules raise broader health concerns (Georgiou et al., 2026; Wang et al., 2019). Current evidence favors a conditional rather than uniformly positive or negative view of gaming, with outcomes varying across participation characteristics and individual and environmental factors (Bediou et al., 2018; Poulus et al., 2026; Tang et al., 2026). The central question is increasingly not whether gaming is beneficial or harmful, but for whom, under what conditions, and with what performance–health trade-offs.

## 2 State of the field

Cross-sectional comparisons report selected perceptual and attention-related advantages among experienced gamers, but training studies show mixed evidence for broader cognitive transfer (Bediou et al., 2018; Sala et al., 2018). These differences may reflect practice, pre-existing characteristics, or both, and their translation to meaningful competitive performance remains unclear (Pedraza-Ramirez et al., 2020).

Psychological research addresses both mental-health problems and resources associated with well-being in esports. A scoping review of 89 studies (Poulus et al., 2026) identified positive indicators, including intrinsic motivation, self-efficacy, and psychosocial skills, alongside anxiety and other mental-health problems, with outcomes varying by player experience, coping strategies, and competitive environment. Yet 56.2% of studies were cross-sectional and only 6.7% longitudinal, limiting conclusions about development over time.

Methodologically, esports research complements questionnaires and cognitive tests with physiological and sensor-based measures. A systematic review (Kulkarni and Kuber, 2026) identified 30 studies using heart-rate variability, eye tracking, electrodermal activity, and electroencephalography, combined with machine learning to assess workload, stress, fatigue, expertise, or performance. Small samples, inconsistent validation, limited protocol standardization, and difficulty translating laboratory findings to authentic competition continue to constrain comparability and generalizability (Kulkarni and Kuber, 2026; Pedraza-Ramirez et al., 2025; Welsh et al., 2023).

Although limited and heterogeneous, intervention research is emerging around modifiable psychological processes relevant to performance and wellbeing. A scoping review (Leis et al., 2026) identified only seven psychological intervention studies published between 2022 and 2025, spanning acceptance- and mindfulness-based approaches, coping, arousal reappraisal, and team-focused interventions. A separate systematic review (Wachholz et al., 2026) identified 12 exercise studies, with more consistent effects for acute cognitive and performance-related outcomes than for chronic interventions or direct esports performance. Overall, intervention evidence remains insufficient for firm conclusions about sustained real-world effects (Leis et al., 2026; Poulus et al., 2026; Wachholz et al., 2026).

## 3 Contributions of the Research Topic

The present Research Topic reflects this more conditional and mechanism-oriented view. Ozdayi et al. link passion, pressure vulnerability, and resilience, while Kilci, Bekir et al. examine cognitive flexibility as an indirect pathway between negative problem orientation and social problem-solving. Kilci, Yonal et al. similarly position cognitive flexibility within the association between computational thinking and creative problem-solving. Lee and Oh extend the psychological scope through conditional pathways linking self-compassion, resilience, self-efficacy, and wellbeing. Cui adds a social dimension by showing that dominance-oriented status motives and gendered traits relate differently to in-game aggression. Together, these studies highlight cognitive, motivational, and social processes that may help explain variation in psychological and behavioral outcomes. As most of these designs are observational, their mediation and moderation models should be interpreted as explanatory association models rather than causal mechanisms.

Other contributions make heterogeneity itself a central finding. Van Ruysevelt et al. show that mental-health differences associated with self-identified neurodivergence vary across competitive tiers. Liu et al. report positive associations between participation in multiplayer online battle arena esports and self-efficacy, social support, and trait sport confidence among individuals with disabilities, alongside U-shaped relationships between daily participation duration and both self-efficacy and trait sport confidence. In over 15,000 professional players, Kang finds no aggregate relative-age effect after accounting for birth seasonality in the general population, while identifying cohort-specific patterns and an indirect association between birth quarter and earnings through debut age. Together, these studies highlight the role of psychological resources to affect adaptation and wellbeing. They also caution against generalizing findings across player groups, participation levels, or developmental contexts and against assuming that greater esports participation necessarily produces greater psychological benefits.

The Research Topic also qualifies broad claims about cognitive and performance benefits and extends into related digital and virtual contexts. Pasescu et al. find no significant associations between gaming frequency and visual or auditory spatial ability, reinforcing caution against universal claims of cognitive benefits. Wang and Sun examine music as a potential regulator of arousal, attention, cognitive load, and rhythmic timing in esports, while emphasizing important boundary conditions and limited direct esports evidence. Outside esports-specific samples, Xie et al. find in a randomized trial that open-skill exercise produced greater improvements in ecological executive function and mobile-phone dependence than closed-skill exercise among adolescents with mobile-phone dependence. Separately, Festino et al. find that non-immersive virtual environments enhanced enjoyment and selected dimensions of flow during cycling without altering exercise performance.

Methodologically, the Research Topic spans surveys, repeated measurement, randomized interventions, ecologically oriented behavioral assessments, large-scale career data, virtual environments, and naturalistic machine-learning analyses. Kadri et al. add an especially naturalistic approach by applying machine learning to brief pre-match voice recordings from professional Counter-Strike communication. Together, the Research Topic connects conventional self-report research with experimental, large-scale, virtual, and naturalistic approaches.

## 4 Outlook

Three priorities emerge. First, research should move beyond cross-sectional associations toward evidence on causality, development, and modifiability. Theory-driven models should examine how cognitive, motivational, affective, and social processes interact over time rather than treating psychological constructs in isolation (Pedraza-Ramirez et al., 2025). Longitudinal studies should distinguish characteristics that develop through gaming and esports participation from pre-existing characteristics and reciprocal processes (Pedraza-Ramirez et al., 2020). Experimental and intervention studies should test modifiable factors and sustained effects on wellbeing, health, participation, and competitive performance rather than only short-term surrogate outcomes (Leis et al., 2026; Poulus et al., 2026; Wachholz et al., 2026).

Second, measured constructs need stronger links to real-world functioning. Self-report, cognitive, physiological, digital, and sensorimotor measures should be integrated with objective competitive performance and meaningful health outcomes (Kulkarni and Kuber, 2026; Pedraza-Ramirez et al., 2025; Poulus et al., 2026). Research should establish whether these measures explain or predict such outcomes rather than add measurement complexity (Kulkarni and Kuber, 2026; Welsh et al., 2023).

Third, heterogeneity should be treated as a primary research question. Future studies should determine whether relationships differ by competitive level, game genre, developmental stage, participation intensity, and demographic or social context (Pedraza-Ramirez et al., 2025; Poulus et al., 2026). Consistent definitions and direct subgroup comparisons would improve comparability and identify where domain-specific models are needed. Sensorimotor processes, women, gender-diverse individuals, and non-Western populations remain insufficiently studied (Dupuy et al., 2024; Pedraza-Ramirez et al., 2025; Poulus et al., 2026).

Increasing professionalization also raises questions about sustainable careers. Research and support structures should address dual-career pathways combining competition with education or employment and preparation for post-competitive transitions (Hong and Connelly, 2022; Zhao et al., 2024).

## 5 Conclusion

The field is moving from broad claims about gaming effects toward a more differentiated performance–health framework. The present contributions reinforce that shift by identifying conditional associations, examining psychological pathways, broadening populations and contexts, and adopting more ecologically relevant methods. The next step is to establish how these relationships develop over time, which are causal, what factors are modifiable, and how changes translate into sustainable performance, health, and participation. More integrated psychological perspective may help clarify how individual characteristics and contextual factors shape these outcomes.
